# The Oil of Turpentine in the Treatmen of Neuralgia, and Particularly of Sciatica

**Published:** 1861-04

**Authors:** 


### *The Oil of Turpentine in the Treatment of Neuralgia and Particularly of Sciatica.*

Turpentine was employed many ages ago in the treatment of diseases of the neves. It was used by Galen in the form of a plaster. Sculter exhibited it successfully in wounds of the nerves; and Bonner had even the good fortune to cure a patient of neuralgia by the essential oil of Turpentine; but Archibald first brought it into notice as a remedy for Sciatica. Having informed Cheyne of the success with which he had employed it in this disease, the latter recommended it to Home, who afterwards published in his "Experiments and Facts," seven cases in confirmation of the practice. Since that period, Tupentine has been employed in the above mentioned diseases by many physicians of different countries.

Little benefit is to be expected from the employment of the Oil of Turpentine without due attention to the mode of administering it. It has been exhibited in various proportions, and in very different ways, but we decidedly prefer giving it internally, in small does of twenty drops, three times a day, in order that its absorption may be more gradually but thoroughly effected.

In larger doses it is liable to occasion diarrhœa, by which its peculiar properties are rendered unavailing.

The Oil of Turpentine, when thus given in the above doses, and in some proper vehicle, such as honey, syrup, &c., produces a strong sensation of heat in the stomach, and whole intestinal tube, as well as in the diseased nerve and limb, and sometimes, it even occasions a general sweat. In certain individuals, it causes a colic or diarrhœa, and more rarely, either a dysury or an increased flow of urine. But when too large a dose is given intense colic and diarrhœa, and even vomiting supervene. Yet these formidable signs of irritation, both of the digestive and urinary organs, generally disappear as soon as the medicine is intermitted. In patients whose stomach and bowels are irritable, a small quantity of opium is found a useful addition to the Turpentine, by moderating its stimulating effects on the mucous membrane of those parts.

When the Oil of Turpentine is administered in the manner and quantity just described, it would seem to be particularly powerful in the removal of Sciatica. Be this as it may, its efficacy is also remarkable for curing other species of neuralgia which affect the extremities.

When we attempt to deduce from the phenomena which follow the exhibition of the oil, the mode of its operation and the cause of its being efficacious, we cannot refer the latter to its purgative, its diuretic, or its sudorific effects: since this augmentation of the different secretions is neither regular nor constant in its occurrence, and never bears any proportion to the benefits derived by the patient. Besides, we daily see patients who are purged, sweated, &c., much more abundantly, by other medicines, without deriving the least benefit; and it was this reflection which led Home to attribute to the Oil of Turpentine a specific influence over Sciatica.

Some physicians have supposed that this medicine produces its sanitary effects on the nervous system by causing a revulsion from the brain to the stomach and skin. Others, on the contrary, attribute its efficacy to a revulsion on the nerves, which is sympathetic with that of the stomach.—

Others, however, conceive that the stimulation which this oil communicates to the mucous membrane of the stomach is equally produced in the nerves affected, and to a greater or less degree in proportion as they are more or less morbidly affected. Which, in my opinion, serves to explain the fact that this medicine is more efficacious in severe and obstinate cases, than in mild and recent ones of neuralgia. The new modification which is thus effected in the state of the nerve would seem, therefore, to dispose it to resume its natural action, that of health.

The heat which those persons who are either cured or relieved, feel in the affected parts, seems to confirm the explanation adopted. As to the question, whether the Turpentine acts directly on the nerves by absorption, or exerts its influence over them indirectly and sympathetically, we are inclined to adopt the first of these hypotheses; and we found our opinion on the fact that this Oil is nearly always reported to fail in curing those cases of neuralgia where it produces violent purging, which is also true in respect to all other substances employed in this disease, and whose only effect is to irritate the mucous membrane of the stomach and intestines.

Its action on the urinary organs would appear to be seldom useful, on the contrary, often injurious. This medicine is of efficacy in all cases of neuralgia affecting the extremities, and particularly in Sciatica, when this disease is simple in its character and evinces no sign that the nerve is either altered in its structure, in a state of inflammation or compressed by the formation of a contiguous tumor.

The chance of cure by this medicine is greatest, when the pain is so intense as to indicate distinctly the course of the nerve, and so obstinate in its nature as to yield to no other treatment whatever.

TEREBINTH.

[*Med. Jour. of N. C.*

